# Doxorubicin‐induced skeletal muscle atrophy: Elucidating the underlying molecular pathways

**DOI:** 10.1111/apha.13400

**Published:** 2019-10-31

**Authors:** Anouk E. Hiensch, Kate A. Bolam, Sara Mijwel, Jeroen A. L. Jeneson, Alwin D. R. Huitema, Onno Kranenburg, Elsken van der Wall, Helene Rundqvist, Yvönne Wengstrom, Anne M. May

**Affiliations:** ^1^ Julius Center for Health Sciences and Primary Care University Medical Center Utrecht Utrecht University Utrecht The Netherlands; ^2^ Department of Neurobiology, Care Sciences and Society Karolinska Institutet Stockholm Sweden; ^3^ Neuroimaging Centre Division of Neuroscience University Medical Center Groningen Groningen The Netherlands; ^4^ Department of Radiology Academic Medical Center Amsterdam University of Amsterdam Amsterdam The Netherlands; ^5^ Department of Pharmacy & Pharmacology The Netherlands Cancer Institute‐Antoni van Leeuwenhoek and MC Slotervaart Amsterdam The Netherlands; ^6^ Department of Clinical Pharmacy University Medical Center Utrecht University Utrecht The Netherlands; ^7^ UMC Utrecht Cancer Center University Medical Center Utrecht Utrecht The Netherlands; ^8^ Department of Medical Oncology University Medical Center Utrecht Utrecht University Utrecht The Netherlands; ^9^ Department of Cell and Molecular Biology Karolinska Institutet Stockholm Sweden; ^10^ Theme Cancer Karolinska University Hospital Stockholm Sweden

**Keywords:** doxorubicin, mitochondrial dysfunction, muscle atrophy, reactive oxygen species, skeletal muscle, ubiquitin‐proteasome pathway

## Abstract

**Aim:**

Loss of skeletal muscle mass is a common clinical finding in cancer patients. The purpose of this meta‐analysis and systematic review was to quantify the effect of doxorubicin on skeletal muscle and report on the proposed molecular pathways possibly leading to doxorubicin‐induced muscle atrophy in both human and animal models.

**Methods:**

A systematic search of the literature was conducted in PubMed, EMBASE, Web of Science and CENTRAL databases. The internal validity of included studies was assessed using SYRCLE’s risk of bias tool.

**Results:**

Twenty eligible articles were identified. No human studies were identified as being eligible for inclusion. Doxorubicin significantly reduced skeletal muscle weight (ie EDL, TA, gastrocnemius and soleus) by 14% (95% CI: 9.9; 19.3) and muscle fibre cross‐sectional area by 17% (95% CI: 9.0; 26.0) when compared to vehicle controls. Parallel to negative changes in muscle mass, muscle strength was even more decreased in response to doxorubicin administration. This review suggests that mitochondrial dysfunction plays a central role in doxorubicin‐induced skeletal muscle atrophy. The increased production of ROS plays a key role within this process. Furthermore, doxorubicin activated all major proteolytic systems (ie calpains, the ubiquitin‐proteasome pathway and autophagy) in the skeletal muscle. Although each of these proteolytic pathways contributes to doxorubicin‐induced muscle atrophy, the activation of the ubiquitin‐proteasome pathway is hypothesized to play a key role. Finally, a limited number of studies found that doxorubicin decreases protein synthesis by a disruption in the insulin signalling pathway.

**Conclusion:**

The results of the meta‐analysis show that doxorubicin induces skeletal muscle atrophy in preclinical models. This effect may be explained by various interacting molecular pathways. Results from preclinical studies provide a robust setting to investigate a possible dose‐response, separate the effects of doxorubicin from tumour‐induced atrophy and to examine underlying molecular pathways. More research is needed to confirm the proposed signalling pathways in humans, paving the way for potential therapeutic approaches.

## 1 INTRODUCTION

Doxorubicin is the most widely used anthracycline cytostatic agent offering the most favourable approach to treating solid tumours and haematological malignancies.^1^ The main anti‐neoplastic effects of doxorubicin include its ability to interfere with the DNA helix and to bind proteins involved in DNA replication and transcription.^1^, ^2^ Ultimately, such interactions result in cell death because of an inhibition of DNA, RNA and protein synthesis. However, because of this non‐specific mechanism of action, healthy cells with a high proliferative potential are also affected. As a consequence detrimental side effects, including nausea, hair and weight loss, fatigue, cardiotoxicity and skeletal muscle atrophy, presently limit the clinical use of higher and more effective doses of doxorubicin when administered systemically.^1^, ^2^, ^3^ Strategies to resolve this problem would entail either local delivery of the drug to tumours^4^ or concomitant administration of drugs to limit or even prevent unwanted side effects. The latter will require improved understanding of the mechanistic basis of doxorubicin‐induced damage to non‐tumour cells. The present study was undertaken to specifically evaluate and review current knowledge of the relation between doxorubicin treatment and skeletal muscle tissue.

Patients undergoing cytotoxic chemotherapy often experience changes in body mass and composition because of reduced food intake and metabolic changes, including the development of an inflammatory environment, an increased energy expenditure and excess catabolism.^5^ Estimates suggest that up to 80% of patients with cancer experience weight loss.^6^, ^7^ Excessive wasting of skeletal muscle is assumed to contribute to these alterations in body mass. The prevalence of muscle atrophy varies from 14% in patients with early stage breast cancer, to 27% in patients with advanced breast cancer and 55%‐56% in patients with non‐Hodgkin lymphoma. Up to now, the extent to which cytotoxic chemotherapy contributes to skeletal muscle atrophy is not clear. Therefore, the goal of the present study was to determine the extent to which skeletal muscle tissue is impacted by doxorubicin treatment. To date, preclinical studies have shown that chemotherapy alone, independent of neoplastic disease, can promote muscle loss.^8^, ^9^ In patients with cancer with both early and advanced stage disease, the loss of muscle mass negatively affects clinical and patient‐reported outcomes.^10^ It can lead to the loss of muscle strength, progressive functional impairment, increased levels of fatigue and, thereby decreased quality of life.^11^, ^12^ The combination of fatigue and reduced muscle strength is a significant burden for cancer patients and can last up to 10 years following the cessation of chemotherapy.^13^ Furthermore, it has been reported that skeletal muscle loss is a strong prognostic factor for prognosis and drug‐associated toxicity, regardless of body weight loss.^14^, ^15^ Conversely, cancer patients with higher muscle mass generally tolerate higher, and, therefore, more effective doses of chemotherapy, which in turn increases the chance of disease‐free survival.^16^, ^17^

Since it has been suggested that doxorubicin induces skeletal muscle atrophy, it is important to develop a countermeasure to prevent doxorubicin‐induced skeletal muscle atrophy by acquiring a detailed understanding of the mechanisms responsible for this unwanted side effect. While the mechanisms by which cancer induces skeletal muscle atrophy have been extensively studied,^7^, ^11^, ^18^ less is known about the specific effects of doxorubicin on skeletal muscle tissue. Emerging evidence suggests that doxorubicin induces oxidative stress 2, 19, 20, 21, 22 possibly leading to mitochondrial dysfunction, a process closely associated with the activation of proteolytic signalling pathways. Another molecular pathway that seems to be involved in doxorubicin‐induced skeletal muscle wasting is the ubiquitin‐dependent proteasome pathway.^23^ In addition, autophagy has also been put forward as a potential mechanism involved in doxorubicin‐induced protein degradation.^23^, ^24^ However, individual studies have been focusing on particular molecular pathways instead of comparing the relative contribution of these pathways to doxorubicin‐induced skeletal muscle atrophy. Therefore, the exact mechanisms and molecular pathways responsible for doxorubicin‐induced skeletal muscle atrophy remain elusive.

Currently, reports on the effect of doxorubicin on human skeletal muscle tissue are limited. Available data on the effect of doxorubicin on skeletal muscle tissue and the above mentioned molecular pathways are mainly obtained from animal models. Although animal models do not replicate all aspects of muscle atrophy in humans, results from preclinical studies do provide a controlled and valuable model to examine the effect of doxorubicin on skeletal muscle and identify the proposed molecular pathways in a cancer and non‐cancer setting.^18^ Furthermore, results from preclinical studies provide a robust setting to investigate a possible dose‐response, separate the effects of doxorubicin from tumour‐induced atrophy and to examine underlying molecular pathways and test potential therapeutic targets.

The aim of this meta‐analysis and systematic review was to evaluate and review current knowledge of the effects of doxorubicin on skeletal muscle tissue, in terms of skeletal muscle weight, muscle fibre cross‐sectional area (CSA), and muscle strength, and to provide insight into the underlying molecular mechanisms behind these effects. First, we quantified the effect of doxorubicin on skeletal muscle (ie skeletal muscle weight and muscle fibre CSA) by conducting a meta‐analysis. Second, we evaluated the findings of studies that assessed muscle strength and the molecular pathways of the effect of doxorubicin on skeletal muscle tissue in both human and animal models. Lastly, we delineated the potential significance of these proposed mechanisms with respect to the development of targeted therapeutic strategies.

## 2 RESULTS

### 2.1 Study selection process

**Figure 1:**
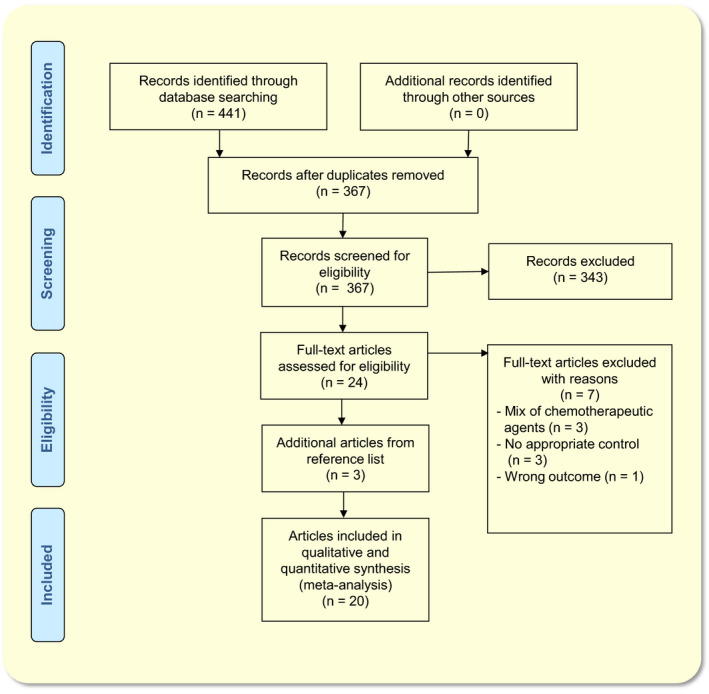
PRISMA Flow Diagram of the study selection process

Details of the study selection process are depicted in a flow chart (Figure 1). The search strategy retrieved 367 unique records. The selection procedure resulted in 20 eligible articles.

### 2.2 Study characteristics

Details of the study characteristics (eg strain, dosing schedule and concentration) are shown in Table 1. Studies conducted in humans were not identified as being eligible because of the absence of an appropriate control group. All eligible articles involved studies conducted in rodents without tumours, of which 9 were in mice and 11 in rats. Data were predominantly obtained in males (75%). A dose of 20 mg/kg in rodents is in the range of the commonly used human dose when scaled according to the commonly applied methods.^25^, ^26^ Nine studies used this dose (45%), whereas various lower doses were used in the other studies. The majority of studies (75%) examined the short‐term effects (20‐72 hours) of doxorubicin on skeletal muscle, whereas six studies examined the long‐term effects (5 days to 4 weeks, which is equivalent to 2‐11 human years^27^) of doxorubicin.

**Table 1:** Characteristics of included studies

Study ID	Animal characteristics			Study characteristics			Intervention characteristics		
Reference	Species	Sex	Age	Experimental groups (n)	Control groups (n)	Origin skeletal muscle sample	Dose (mg/kg)	Number of doses	Acute or long‐term effect of doxorubicin
Dirks‐Naylor et al 2013	F344/Crl rats	M	6 weeks	1. Doxorubicin (n = 8)	1. Vehicle control (NaCl) (n = 7)	Soleus	20	Single dose	Acute (72 h)
Gilliam et al 2009	C57BL/6 and TNFR‐1 receptor deficient mice	M	6‐8 weeks	1. Doxorubicin (n = 6)	1. Vehicle control (PBS) (n = 5)	EDL	20	Single dose	Acute (72 h)
				2. Doxorubicin in TNFR‐1 receptor deficient mice (n = 8)					
Gilliam et al 2011^a,b^	C57BL/6 and TNFR‐1 receptor deficient mice	M	6‐8 weeks	1. Doxorubicin (n = 5)	1. Vehicle control (PBS) (n = 6)	Diaphragm	20	Single dose	Acute (72 h)
Gilliam et al 2013	Sprague Dawley rats	M	8‐10 weeks	1. Doxorubicin (n = 8)	1. Vehicle control (PBS) (n = 8)	Gastrocnemius	20	Single dose	Acute (72 h)
Gilliam et al 2016	C57BL/6N mice	F	12 weeks	1. Doxorubicin (n = 10)	1. Vehicle control (PBS) (n = 10) 2. MCAT control (n = 12)	Soleus	20	Single dose	Acute (72 h)
				2. Tumour bearing (n = 10)					
				3. Doxorubicin + tumour bearing (n = 9)					
				4. MCAT doxorubicin (n = 11)					
				5. MCAT tumour bearing (n = 10)					
				6. MCAT doxorubicin + tumour bearing (n = 10)					
Grabowiecki et al 2015	Mice	•	8 weeks	1. Doxorubicin (n = 5‐12)	1. Vehicle control (n = 5‐12)	Gastrocnemius and tibialis	18	Single dose	Long (15 days)
				2. Doxorubicin + AGT251 (n = 5‐12)					
Huang et al 2016	Sprague Dawley rats	•	4‐5 months	Exercise challenge and survival experiment:	Exercise challenge and survival experiment:	Soleus	2.5	Exercise: single dose	Exercise: Acute (24 h)
				1. Doxorubicin (n = 14)	1. Vehicle control (n = 14)			Survival: Every 3 days	Survival: 40 days
				2. Q10 (n = 14)					
				3. Doxorubicin + Q10 (n = 14)					
Hulmi et al 2017	C57BL/6J mice	M	9‐10 weeks	Long‐term and acute experiment:	Long‐term and acute experiment:	Heart and tibialis anterior	Long‐term: 6	Long‐term: 4, every third day	Long‐term: 2 and 4 weeks
				1. Doxorubicin	1. Vehicle control (PBS)		Acute: 15	Acute: Single dose	Acute: 20 h
				2. Doxorubicin + sACVR2B‐Fc					
Hydock et al 2011	Sprague Dawley rats	M	•	1. Doxorubicin dose 1 (n = 7)	1. Vehicle control (saline) (n = 7)	Heart, soleus and EDL	1. 10	Single dose	Acute (120 h)
				1. Doxorubicin dose 2 (n = 7)			2. 12.5		
				1. Doxorubicin dose 3 (n = 7)			3. 15		
Kavazis et al 2014	Sprague Dawley rats	M	6 months	1. Sedentary doxorubicin (n = 6)	1. Sedentary vehicle control (saline) (n = 6)	Soleus	20	Single dose	Acute (24 h)
				2. Exercise doxorubicin (n = 6)	2. Exercise vehicle control (n = 6)				
Lima Junior et al 2016	Wistar rats	M	14 weeks	1. Doxorubicin (n = 13)	1. Vehicle control (saline) (n = 13)	EDL	15	Single dose	Acute (72 h)
Min et al 2014	Sprague Dawley rats	M		1. Doxorubicin (n = 10)	1. Vehicle control (saline) (n = 10)	Diaphragm	2.5	6 alternate days	Long (2 weeks)
				2. Doxorubicin + SMI (n = 10)					
Min et al 2015	Sprague Dawley rats	F	4‐6 months	1. Doxorubicin (n = 8)	1. Vehicle control (saline) (n = 8)	Heart, diaphragm, soleus and plantaris	20	Single dose	Acute (48 h)
				2. Doxorubicin + antioxidant SS31 (n = 8)	2. Control + antioxidant SS31 (n = 8)				
				3. Doxorubicin + calpain inhibitor SJA 6017 (n = 8)	3. Control + calpain inhibitor SJA 6017 (n = 8)				
Nissinen et al 2016	C57BL/6J mice	M	9‐10 weeks	Exp. 1‐4: Doxorubicin (n = 6‐16) and Doxorubicin + sACVR2B (n = 5‐17)	Exp. 1‐4: Vehicle control (PBS) (n = 5‐15)	Tibialis anterior, gastrocnemius and soleus	Exp. 1‐3:6	Exp. 1‐3:4	Exp. 1‐2: Long (2 weeks)
				Exp. 5: LLC + Doxorubicin, LLC + sACVR2B and LLC + Doxorubicin + sACVR2B	Exp. 5: Healthy control and LLC‐ tumour bearing + vehicle		Exp. 4:15	Exp.4: single dose	Exp. 3: Long (4 weeks)
							Exp. 5‐6:12	Exp. 5:2	Exp. 4: Acute (20 h)
									Exp. 5: Long (8 days)
Smuder et al 2011^a,b^	Sprague Dawley rats	M	6 months	1. Sedentary doxorubicin (n = 5)	1. Sedentary vehicle control (saline) (n = 7)	Soleus	20	Single dose	Acute (24 h)
				2. Exercise‐trained doxorubicin (n = 6)	2. Exercise‐trained vehicle control (saline) (n = 6)				
Yu et al 2014	C57BL/6J mice	M	8‐12 weeks	1. Doxorubicin (n = 7)	1. Vehicle control (saline) (n = 7)	Gastrocnemius	15	Single dose	Acute (96 h)
				2. Doxorubicin + unacylated ghrelin (n = 7)	2. Vehicle control + unacylated ghrelin (n = 4)				
				3. Doxorubicin + acylated ghrelin (n = 7)	3. Vehicle control + acylated ghrelin (n = 4)				
Yu et al 2014	C57BL/6J mice	M	8‐12 weeks	1. Doxorubicin (n = 7)	1. Vehicle control (saline) (n = 7)	Gastrocnemius	15	Single dose	Acute (96 h)
				2. Doxorubicin + [D‐Lys‐3]‐GHRP‐6 (n = 4)	2. Vehicle control + [D‐Lys‐3]‐GHRP‐6 (n = 5)				
				3. Doxorubicin + [D‐Lys‐3]‐GHRP‐6 + TAK‐779 (n = 4)					
				4. Doxorubicin + [D‐Lys‐3]‐GHRP‐6 + YIL781 (n = 4)					
				5. Doxorubicin + [D‐Lys‐3]‐GHRP‐6 + AMD3100 (n = 4)					
Zima et al 2001	Wistar rats	M	•	1. Doxorubicin 2.5 h (n = 5‐8)	1. Vehicle control (saline) 2.5 h (n = 5‐8)	Gastrocnemius	5	Single dose	Acute (24 h and 2.5 h)
				2. Doxorubicin 24 h (n = 5‐8)	2. Vehicle control (saline) 24 h (n = 5‐8)				
				3. Doxorubicin + ICRF‐187 2.5 h (n = 5‐8)	3. Vehicle control + ICRF‐187 2.5 h (n = 5‐8)				
				4. Doxorubicin + ICRF‐187 24 h (n = 5‐8)	4. Vehicle control + ICRF‐187 24 h (n = 5‐8)				

### 2.3 Risk of bias and quality of reporting

**Figure 2:**
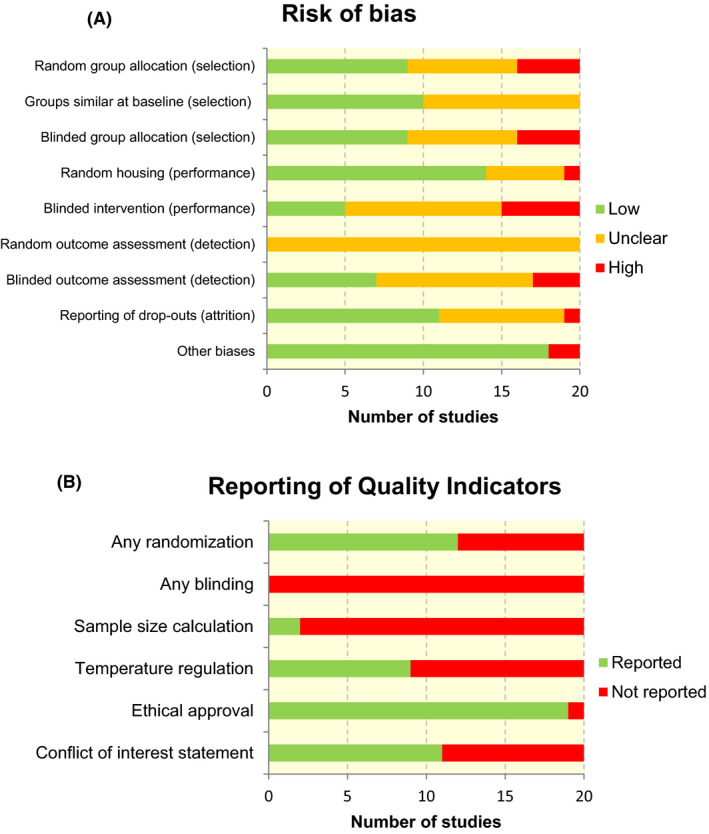
Risk of bias graphs. Graph A displays the risk of selection, performance, detection, attrition and other biases, which were assessed in all included studies using SYRCLE’s risk of bias tool. Graph B displays the reporting of six key quality indicators. Review authors’ judgements about each item are presented as absolute numbers across all included studies

The risk of bias and study quality assessment of individual studies is presented in Data S3. When assessing the individual components of the SYRCLE’s risk of bias tool, inadequate reporting of the methodology often led to an unclear risk of bias (Figure 2A). Of the three entries that are related to selection bias, random and blinded group allocation were most poorly reported. Originally, the details of methods used for both randomization and blinding of the outcome assessor were unclear in all included articles. Only after contacting the authors, these methods were clarified for nine studies. Studies that matched vehicle control and intervention groups on important baseline characteristics were indicated as low risk of selection bias. All authors failed to report details on measures to reduce performance and detection bias. Again, after contacting the authors, these details were clarified.

The overall reporting of quality indicators for the 20 included studies is presented in Figure 2B. For 12 (60%) studies, it was reported that the experimental groups were randomized in some way. Blinding of the experiment at any level was not reported in any of the studies. In only two studies (10%), a sample size calculation was reported to support the chosen group sizes. Approximately half of the studies (45%) reported that the temperature of the animal room was regulated within a physiological range. In the majority of studies (95%), a statement of ethical approval was reported. A conflict of interest statement was present in 11 studies (55%), in which the authors of two of these studies reported a possible conflict of interest.

### 2.4 Effects of doxorubicin on muscle mass and cross‐sectional area

**Figure 3:**
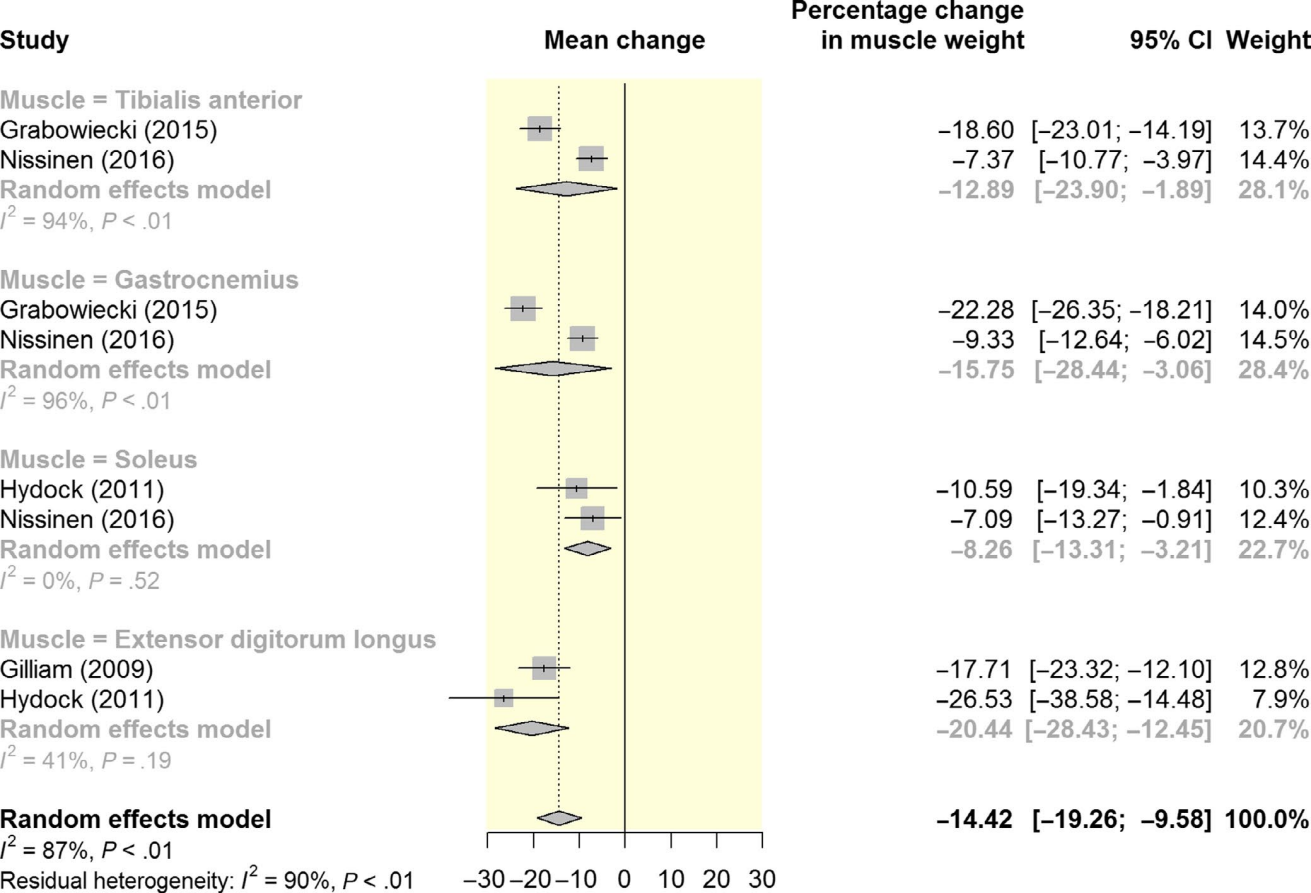
Forest plot of meta‐analysis estimates of the effect of doxorubicin on skeletal muscle weight (gram). Results are presented as percentage change in muscle weight with accompanying 95% CI. Subgroup analyses were conducted to assess the effect of doxorubicin on specific limb muscles

In general, doxorubicin administration resulted in a significant decrease in body weight, however, because of the heterogeneity in reporting, specifics cannot be concluded.^13^, ^19^, ^28^, ^29^, ^30^, ^31^, ^32^, ^33^, ^34^ In one study, the reduced body weight was sustained for 4 weeks after the cessation of chemotherapy.^28^ The decrease in body weight was accompanied by a reduction in skeletal muscle weight and a decrease in lean body mass and fat mass.^13^, ^19^, ^28^, ^31^, ^32^, ^34^, ^35^, ^36^ Our meta‐analysis revealed that doxorubicin treatment significantly reduced skeletal muscle weight by 14% (95% CI: 9.9 to 19.3) compared to vehicle control (Figure 3). The negative effect of doxorubicin on skeletal muscle weight was most pronounced in the extensor digitorum longus muscle compared to other limb muscles.

**Figure 4:**
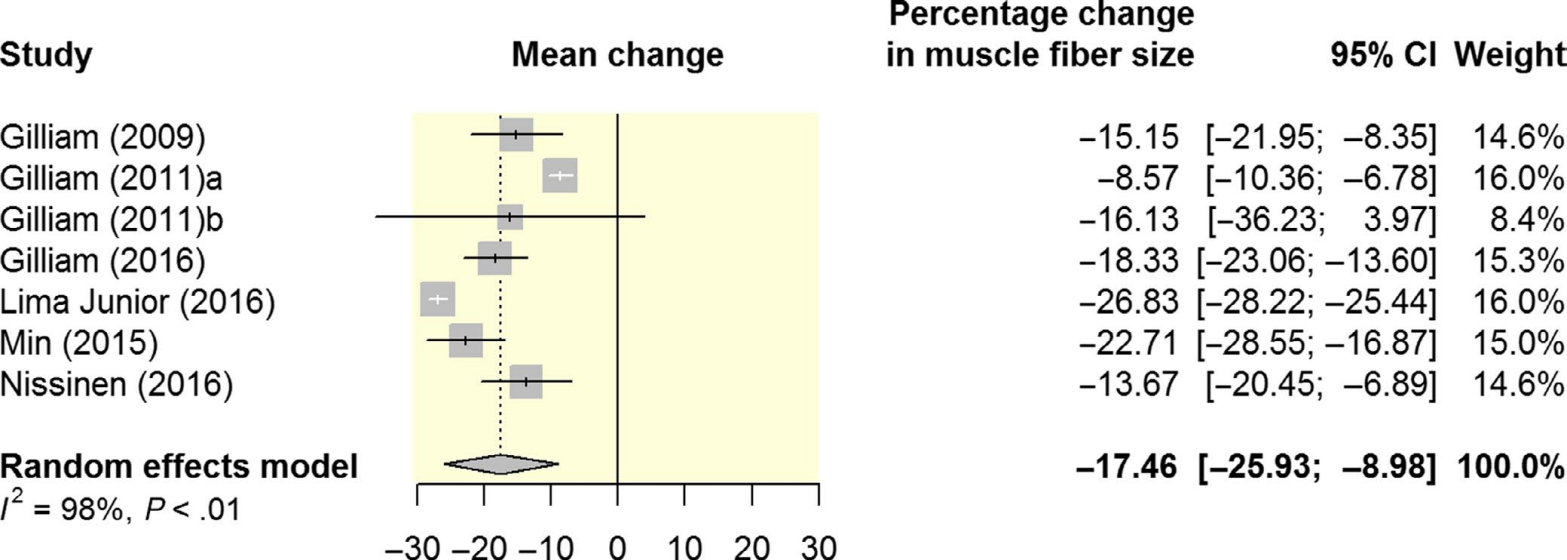
Forest plot of meta‐analysis estimates of the effect of doxorubicin on muscle fibre CSA (μm^2^). Results are presented as percentage change in muscle fibre size with accompanying 95% CI

In line with reduced skeletal muscle weight, treated animals tended to have a decreased muscle fibre CSA.^13^, ^21^, ^23^, ^28^, ^29^, ^30^, ^31^, ^37^ Specifically, doxorubicin administration in rats resulted in a significant decrease in type I muscle fibre CSA (slow twitch oxidative; SO), type IIa (fast twitch oxidative glycolytic; FOG) and type IIx/b muscle fibre CSA (fast twitch glycolytic; FG) in the diaphragm, plantaris and soleus muscles.^21^ Our meta‐analysis demonstrated that doxorubicin administration significantly reduced muscle fibre CSA (μm^2^) by 17% (95% CI: 9.0 to 26.0; 7 studies) when compared to vehicle controls (Figure 4). Yu et al (2014 and 2015) assessed the gastrocnemius muscle fibre size by measuring the feret diameters of the fibres. Both studies did not observe a significant difference in the mean fibre size.^23^, ^38^

Parallel to negative changes in muscle mass, muscle strength was even more decreased in response to doxorubicin administration. Gilliam et al (2011) showed that doxorubicin treatment exacerbated diaphragm dysfunction, since maximal absolute force was depressed with 50%‐60% in doxorubicin‐treated animals.^37^ When normalized for muscle fibre CSA, specific force remained depressed. These findings are in line with three other studies demonstrating that doxorubicin administration at clinical doses results in skeletal muscle weakness, as shown by the decrease in muscle‐specific force.^21^, ^31^, ^39^ Hydock et al (2011) observed that decrements in skeletal muscle function, measured as maximal twitch force, were dose dependent.^32^ Doxorubicin treatment resulted in a 45%, 60% and 74% reduction in maximal twitch force of the soleus in rats receiving injections of 10, 12.5 or 15 mg/kg respectively. Correspondingly, Ge et al (2014) reported a significantly lower force‐frequency relationship in doxorubicin‐treated animals compared to the control animals, indicating an impaired muscle function.^40^

## 3 POTENTIAL MOLECULAR MECHANISMS OF DOXORUBICIN‐INDUCED MUSCLE ATROPHY

Muscle atrophy occurs as a result of increased protein degradation as well as from decreased protein synthesis. Increased protein degradation is mainly driven by oxidative stress, autophagy and activation of the ubiquitin‐proteasome pathway, whereas reduced protein synthesis is most likely driven by an altered response to growth‐promoting pathways. However, the exact molecular mechanisms behind doxorubicin‐induced muscle atrophy are currently not fully elucidated.

### 3.1 Oxidative stress induced by mitochondrial dysfunction following doxorubicin administration

The majority of skeletal muscle cells are terminally differentiated and may not be significantly affected by the main anti‐cancer effect of doxorubicin (ie inhibition of DNA replication). Instead, doxorubicin is suggested to induce atrophy partly through causing mitochondrial dysfunction in skeletal muscle.^2^, ^19^ Initially, chemotherapeutic agents have the potential to attenuate mitochondrial respiration.^41^ Gilliam et al (2013) and Gilliam et al (2016) evaluated mitochondrial function in permeabilized fibre bundles from the gastrocnemius and soleus muscle, respectively, and observed a rapid (ie within 2 hours) decrease in mitochondrial respiratory capacity following a single doxorubicin injection (20 mg/kg), resulting in impaired electron transport.^13^, ^19^ In these studies, mitochondrial respiration was repressed through the reduction of respiration supported by complex I (pyruvate/glutamate) and complex II (succinate) substrates^13^, ^19^ and through the increased production of H_2_O_2_ (~52%) by the mitochondrial respiratory chain in skeletal muscle.^19^ Interestingly, cancer combined with doxorubicin treatment diminished the effects of either treatment or neoplastic disease alone, resulting in the production of H_2_O_2_ comparable to vehicle controls.^13^ A study from Min *et al* (2015) confirmed that doxorubicin administration (ie single injection of 20 mg/kg) alone results in a decreased mitochondrial respiratory capacity and increased mitochondrial uncoupling and dysfunction, when measured in permeabilized muscle fibres of the diaphragm, soleus and plantaris muscles.^21^ This disruption in electron flow is a potential source of reactive oxygen species (ROS) production. Furthermore, doxorubicin administration reduced the respiratory control ratio (RCR),^21^ which is an indicator of mitochondrial uncoupling and dysfunction.^42^ These findings suggest that mitochondria are a major source of (mitochondrial) ROS formation in skeletal muscle in response to doxorubicin.^13^, ^19^

Reactive oxygen species are oxygen‐derived molecules that can cause damage to DNA and proteins if ROS levels are dramatically increased and not neutralized by antioxidants.^43^ ROS, such as hydrogen peroxide (H_2_O_2_), are the normal products of metabolism and involved in multiple cellular signalling pathways. However, increased levels induced by environmental stress or reduced antioxidant activity may lead to oxidative stress, which in turn results in oxidative damage.^43^

Reactive oxygen species are the potential mediators of chemotherapy‐induced skeletal muscle atrophy, and able to contribute to muscle atrophy both directly (ie through oxidative damage) and indirectly (ie through redox signalling in proteolytic pathways).^11^ In response to the oxidative stress that is induced by the above‐mentioned mechanisms, lipid peroxidation occurs and biologically active aldehydes are produced, including 4‐hydroxy‐2‐nonenal (4‐HNE).^21^ Additionally, 4‐HNE can induce oxidative damage by forming adducts with muscle proteins. These 4‐HNE protein conjugates can be measured as a biomarker of lipid peroxidation to determine oxidative modification of muscle proteins. Two studies demonstrated that doxorubicin administration resulted in significantly increased levels of 4‐HNE modified proteins.^21^, ^44^

While ROS levels have been shown to be elevated following doxorubicin administration, the levels of heat shock proteins (HSPs) have been shown to decrease in the soleus muscle, indicating a reduced capacity to protect the muscle fibre against oxidative stress.^44^ HSPs play a role in protein synthesis and have been shown to protect cells from protein damaging stressors (eg mitochondrial production of ROS).

**Figure 5:**
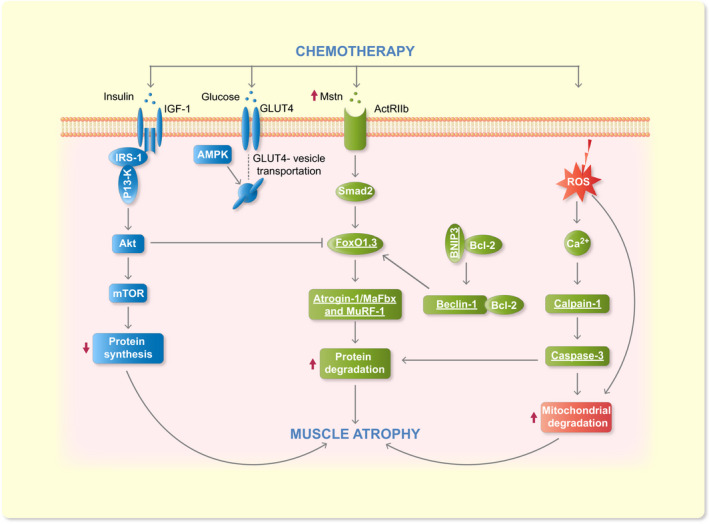
A proposed schematic diagram of signalling pathways for doxorubicin‐induced muscle atrophy. Many intracellular pathways participate in doxorubicin‐induced muscle atrophy. The pathways are divided in three main pathways in this diagram: (a) The disrupted insulin signalling pathway leading to decreased protein synthesis, indicated in blue; (b) The autophagic signalling and ubiquitin‐proteasome proteolysis pathway leading to increased protein degradation, indicated in  green; and (c) Oxidative stress leading to mitochondrial degradation, indicated in  red. Insulin‐like growth factor 1 (IGF‐1) normally stimulates protein synthesis through Akt and mTOR. The insulin signalling pathway is disrupted in doxorubicin‐induced muscle atrophy and the expression of important proteins (ie GLUT4 and AMPK) involved in glucose uptake is decreased, which results in decreased protein synthesis. Myostatin (Mstn) increases protein degradation by activating forkhead (FOXO) family transcription factors. This allows for the increased transcription of important atrophy‐related genes (ie atrogin‐1/MaFbx and MuRF‐1). Furthermore, mitochondrial respiration is negatively affected by doxorubicin, resulting in excess ROS production. On the one hand this results in the activation of calpain‐1 and caspase‐3, which are proteases that are capable of, respectively, promoting muscle atrophy by cleaving structural proteins and degrading intact myofibrillar proteins. The activity of these two proteases is increased following doxorubicin administration, leading to proteolysis. On the other hand, it results in mitochondrial degradation, which leads to skeletal muscle atrophy. Note that underlined proteins are upregulated in response to chemotherapy

These findings suggest that mitochondrial respiration is negatively affected by doxorubicin, resulting in excess ROS production and compromising the ability to maintain a normal redox state of structural and functional proteins in skeletal muscle. It can be speculated that the mitochondrial production of ROS is a major contributor to doxorubicin‐induced skeletal muscle atrophy (Figure 5; red pathway).

### 3.2 Activation of proteolytic signalling pathways in skeletal muscle

In addition to increased mitochondrial ROS formation, doxorubicin is also capable of (in)directly increasing the activity of several proteolytic pathways in skeletal muscle.^41^ Specifically, increased activity of calpain and caspase and the activation of both the autophagic signalling pathway and ubiquitin‐proteasome pathway have been described to be possibly involved in doxorubicin‐induced muscle atrophy.

#### 3.2.1 Calpain and caspase

Calpain‐1 and caspase‐3 are proteases that are capable of, respectively, promoting muscle atrophy by cleaving structural proteins and degrading intact myofibrillar proteins.^44^ Two studies showed that doxorubicin administration resulted in an increased calpain‐1 and caspase‐3 activity in soleus muscle.^21^, ^44^ Moreover, doxorubicin‐mediated mitochondrial ROS production can potentially also promote this proteolysis via oxidative modification of myofibrillar proteins.^44^ Smuder *et al* (2010) found that oxidative modification increases the susceptibility of myofibrillar proteins to degradation via calpain‐1 and caspase‐3.^22^ In this study, myosin, actin, troponin I as well as α‐actinin were shown to be more susceptible to degradation by calpain‐1 and −2 following oxidation.^22^ In a study of Yu *et al* (2014) a similar trend of the change in caspase‐3 activity was observed, however, the difference between intervention and control did not reach significance.^23^

In addition to alterations in mitochondrial function and protein modifications, chemotherapy can also induce myonuclear DNA damage and subsequent calpain‐1 and caspase‐3 activation leading to apoptosis. The DNA damaging effect in skeletal muscle was confirmed by the increase in the number of TUNEL‐positive nuclei in skeletal muscle in response to doxorubicin treatment.^21^, ^23^, ^24^ The number of TUNEL‐positive nuclei can be used as an index of apoptosis.

The increase in the number of TUNEL‐positive nuclei was accompanied with elevated apoptotic DNA fragmentation^23^ and the abundance of both pro‐apoptotic Bax and anti‐apoptotic Bcl‐2 proteins were significantly increased in the soleus muscle of doxorubicin‐treated mice compared to vehicle control.^23^ The authors speculate that this increase in the protein content of Bcl‐2 might serve as a self‐defence mechanism of skeletal muscle tissue against doxorubicin exposure.

Dirks‐Naylor *et al* (2013) aimed to determine the effects of doxorubicin administration on proteome lysine acetylation status, an indicator of the apoptotic environment, and the expression of various caspases involved in the initiation of apoptosis. In contrast to aforementioned studies, doxorubicin did not affect the expression and activation of caspases.^45^ The differences in results may be attributable to differences in experimental design.

#### 3.2.2 Autophagy

Autophagy is a highly regulated process that can be divided into six different stages: initiation, nucleation, elongation, closure, maturation and degradation.^46^ On the one hand, it removes damaged organelles and protein aggregates to maintain cell survival, while, on the other hand, upregulated autophagy activity can induce apoptosis‐mediated cell death. Smuder and colleagues hypothesized that doxorubicin administration increases the expression of autophagy markers in type I specific soleus muscle.^24^

The initial step in the autophagic signalling pathway involves the formation of an isolation membrane. This membrane will form into a mature autophagosome by the recruitment of proteins. This process is regulated by Atg proteins. The autophagosome initiation protein, Beclin‐1, plays a key role in autophagy by localizing Atg proteins to the isolation membrane.^46^ Smuder *et al* (2011) found that both Beclin‐1 mRNA and protein levels were elevated in the soleus muscle of doxorubicin‐treated mice.^24^

Furthermore, formation of the autophagosome involves interaction of other Atg proteins. Specifically, Atg12, Atg7, Atg4 and LC3 play important roles in the maturation process of the autophagosome.^24^ Findings indicate that Atg12 mRNA and protein levels, levels of the Atg12‐Atg5 complex, protein levels of Atg7 and LC3 mRNA expression levels were significantly increased in the soleus muscle following doxorubicin administration compared to the vehicle control group.^24^ In addition, the ratio of LC3 II‐to‐LC3 I was assessed. This ratio is an indicator of the formation of autophagosomes and commonly used as a marker of autophagy. The LC3II/LC3I was increased in the doxorubicin group compared to vehicle control.^24^

In contrast, Yu *et al* (2014) demonstrated that the expression of autophagic markers in gastrocnemius muscle remained unchanged after 5 days following doxorubicin administration.^23^ This inconsistency can be attributed to the variation of experimental period (5 days in the study of Yu vs 24h after doxorubicin administration in the study of Smuder). Therefore, Yu *et al* (2014) conducted an additional experiment to examine the activation of autophagic signalling at 24h and observed that autophagic signalling was activated at this time point as indicated by the ratio of LC3 II‐to‐LC3 I.^23^ These results suggest that autophagy is upregulated 24h post‐doxorubicin administration and returns to baseline levels 5 days after doxorubicin administration.

During elongation of the isolation membrane, proteins and organelles are sequestered in the cytosol. The autophagosome can fuse with lysosomal vesicles to form the autolysosome where cytoplasmic contents of the autophagosome can be degraded. Cathepsin B, D and L function as lysosomal proteases and are highly expressed in tissues with high protein turnover rates during muscle atrophy.^24^ Data reveal that cathepsin L mRNA and protein expression is increased in the soleus muscle following doxorubicin treatment.^24^ In contrast, the muscle levels of cathepsin B and D mRNA were not increased.

Another study showed that doxorubicin treatment increased the transcription of BNIP3 in soleus muscle.^47^ This result was consistent with the previous studies indicating that doxorubicin induces autophagy in skeletal muscle. BNIP3, a FoxO target gene, is a pro‐apoptotic BH3‐only protein that induces autophagy and promotes mitochondrial dysfunction. Under normal conditions, the protein Beclin‐1 forms a complex with Bcl‐2. However, Bcl‐2 releases Beclin‐1 during stressful conditions in order to bind to BNIP3 to promote autophagy. As a consequence, increased expression of BNIP3 can promote autophagy and apoptosis in skeletal muscle.^47^

Overall, these data suggest that doxorubicin administration induces autophagy through upregulation of several proteins in the autophagic signalling pathway that could promote increased protein breakdown (Figure 5; green pathway).

#### 3.2.3 Ubiquitin‐proteasome pathway

Increased muscle proteolysis occurs mostly through activation of the ubiquitin‐proteasome pathway.^47^ E3 ligases (ie Atrogin‐1/MaFbx and MuRF‐1) in this pathway control polyubiquitination, which is a rate‐limiting step in the ubiquitin‐proteasome proteolysis pathway. E3 ligases are directly involved in skeletal muscle protein breakdown by targeting proteins for degradation.

In addition to contributing to autophagy, FoxO activation can also increase the transcription of atrogenes. Data demonstrate that doxorubicin administration induces muscle‐specific overexpression of Forkhead‐box O1 (FoxO1) and FoxO3 mRNA.^47^ Activation of the forkhead transcription factor family has been shown to be involved in the activation of proteolytic pathways in skeletal muscle through the increased transcription of important atrophy‐related genes (ie atrogin‐1/MaFbx and MuRF‐1), which in turn is regulated by Akt. The study of Kavazis *et al* (2014) revealed that the levels of atrogin‐1/MaFbx and MuRF‐1 mRNA were increased in soleus muscle in sedentary animals following doxorubicin administration.^47^ These results are in line with the findings of Hulmi et al (2017), who showed that E3 ubiquitin ligase atrogin‐1 mRNA was significantly increased by doxorubicin.^36^

The findings of Yu *et al* (2014) on the effects of doxorubicin administration on Akt signalling in skeletal muscle are consistent with the aforementioned studies, showing that doxorubicin has an inhibitory effect on the phosphorylation of Akt in skeletal muscle.^23^

Although previous studies on protein degradation found increased markers of the ubiquitin‐proteasome pathway, the evidence is inconsistent. Nissinen *et al* (2016) also assessed whether doxorubicin‐induced muscle atrophy was because of increased protein degradation by investigating atrophy‐related genes that have previously been shown to be up‐ or downregulated in cancer cachexia. In contrast to other studies, no systematic changes in these genes by doxorubicin were found.^8^, ^28^ Therefore, a gene set enrichment analysis (GSEA) was conducted to detect the small changes in several genes. This analysis revealed a significant increase in the proteolytic pathway and apoptosis, and a trend in the caspase cascade.^28^ Of the individual atrogenes, FoxO1 was the only gene that was significantly induced by doxorubicin.^28^ An explanation as to why Nissinen *et al* (2016) observed only a small upregulation in the protein involved in the proteolytic pathway may be attributed to the dosage. The dosage used (15 mg/kg) was relatively low compared to the ones typically used (20 mg/kg). Therefore, it can be speculated that the mechanisms behind muscle atrophy are dose dependent.

FoxO signalling has also been shown to regulate myostatin expression.^47^ Myostatin is a growth factor that negatively regulates muscle growth and can promote the expression of atrogin‐1/MaFbx through FoxO activation. Kavazis *et al* (2014) showed that the soleus muscle of animals treated with doxorubicin contained higher levels of myostatin mRNA compared to vehicle control^47^ (Figure 5; green pathway). This finding suggests that muscle cell differentiation and regeneration might be impaired.

### 3.3 Decreased protein synthesis

In addition to its potential to induce cellular pathways involved in protein degradation, data also indicate that doxorubicin is capable of decreasing protein synthesis. MAPK signalling is associated with the regulation of muscle size. Nissinen *et al* (2016) reported that the phosphorylation of ERK1/2, a classical MAP kinase, was acutely downregulated in doxorubicin‐treated mice.^28^ A similar decrease in ERK1/2 phosphorylation has been previously reported by Yu *et al* (2014).^23^ However, after 2 and 4 weeks, ERK1/2 phosphorylation levels were again comparable to vehicle control. Nissinen *et al* (2016) also found that muscle protein synthesis was diminished 20 hours following doxorubicin administration, which was revealed by a method called “surface sensing of translation.” No changes because of treatment were observed in the activation of mTORC1 signalling and Akt phosphorylation.^28^ Furthermore, a significant increase in the mRNA expression of REDD1 was found in doxorubicin‐treated animals.^28^ REDD1 is a protein related to muscle atrophy and decreased protein synthesis.^28^ This finding suggests that doxorubicin‐induced REDD1 expression could contribute to decreased protein synthesis.

#### 3.3.1 Insulin signalling pathway

De Lima Junior *et al* (2016) found that doxorubicin administration induced muscle atrophy together with a severe glucose intolerance.^30^ Doxorubicin‐treated animals showed increased levels of glucose, insulin and free fatty acids in serum 72 hours after treatment. The doxorubicin‐treated group showed impaired insulin sensitivity, but the protein expression of insulin receptor (IGF‐1), Akt and PI3‐K did not change in skeletal muscle. Nevertheless, other proteins (ie IRS‐1 and GSK3‐b) involved in the insulin pathway exhibited decreased expression.^30^ Moreover, mRNA and protein levels of GLUT4 and AMPk α were decreased.^30^ It may be that part of the insulin signalling pathway is disrupted in doxorubicin‐induced muscle atrophy, and that the expression of important proteins involved in glucose uptake is decreased, which might result in decreased protein synthesis (Figure 5; blue pathway).

## 4 DISCUSSION

This meta‐analysis showed that doxorubicin treatment caused an average of 14% reduction in muscle weight and an average of 17% reduction in muscle fibre CSA. Parallel to negative changes in muscle mass, muscle strength was also decreased in response to doxorubicin administration. Muscle atrophy can result from both increased protein degradation and decreased protein synthesis.^11^ For each mechanism discussed in this review only a few reports exist. Moreover, strains, doxorubicin concentrations, and dosing schedules differ between these reports; therefore, it is difficult to determine how firmly established a given mechanism is. Based on the current systematic review we can conclude that mitochondrial dysfunction plays an important and central role in doxorubicin‐induced skeletal muscle atrophy. The increased production of ROS plays a key role within this process. Furthermore, the included studies demonstrated that doxorubicin activates all major proteolytic systems (ie calpains, the ubiquitin‐proteasome pathway and autophagy) in the skeletal muscle. Although each of these proteolytic pathways contributes to doxorubicin‐induced muscle atrophy, the activation of the ubiquitin‐proteasome pathway is hypothesized to play a key role. Finally, a limited number of studies investigated the capability of doxorubicin to decrease protein synthesis by a disruption in the insulin signalling pathway.

When comparing the results of this meta‐analysis to other models of skeletal muscle atrophy, we found that a 7‐day hind limb suspension resulted in a comparable decrease in soleus (27.1%) and gastrocnemius muscle (21.5%) weight to body weight ratio in wildtype mice.^48^ Moreover, evidence shows that the network of interacting signalling pathways that we found to be involved in doxorubicin‐induced skeletal muscle atrophy, also share common pathways with disuse atrophy.^49^ Given the similarities between these two models of skeletal muscle atrophy, we hypothesize that disuse might be involved in doxorubicin‐induced skeletal muscle atrophy. Up to now, it is unknown whether rodents receiving chemotherapy move less compared to control rodents. In line with this hypothesis, we can assume that anti‐disuse (physical activity or exercise) might counteract this doxorubicin‐induced atrophy. We will elaborate on this topic in the last paragraph of the discussion.

Studies in this review show that doxorubicin treatment can lead to oxidative stress, autophagy and activation of the ubiquitin‐proteasome pathway. However, it cannot be fully determined whether these effects were because of the direct effects of doxorubicin or because of reduced food and water intake, since most studies did not control for this factor. Interestingly*,* a study by Gilliam and colleagues showed that doxorubicin treatment led to a 50%‐70% reduction in food and water intake 24 hours after drug administration.^31^ Based on this finding, Dirks‐Naylor *et al* (2013) hypothesized that the reduction in lean body mass may in part simply be explained by a reduced food and water intake.^45^ Earlier research has already shown that anorexia stimulates catabolic pathways and proteolysis in skeletal muscle in general. In order to assess the direct effects only, future studies should add an additional group of mice (healthy pair‐fed) to control for any possible effects of reduced food intake by healthy mice resulting from chemotherapy. To do so, healthy mice without chemotherapy should receive the food intake of mice with chemotherapy.

The findings of this systematic review highlight that there are both similarities and differences between studies in the underlying molecular mechanisms found to be induced by doxorubicin. The dose of doxorubicin, the mode of administration, timing of measurement (acute vs long‐term effect in rodents receiving a single chemotherapy dose) and type of muscle studied might play important roles in the identification of doxorubicin‐induced alterations in underlying molecular pathways. The dose of 20 mg/kg of doxorubicin can be assumed to be in the clinical relevant range for humans.^25^, ^26^ However, a lower dose was administered in approximately half of the studies. As a consequence, this might have hampered the comparability of the results. In future studies, the use of this specific dose (ie 20 mg/kg) would enable more stringent comparisons and is recommended to unequivocally establish the relevance of the proposed mechanisms.

### 4.1 Limitations

First, reporting of the methodology of experimental animal studies was typically poor. Indeed, this issue has been previously and repeatedly raised, leaving the present research at substantial risk of bias.^50^ The urgency of this issue was confirmed by the results of the risk of bias assessment, since many items were indicated as “unclear” risk of bias. Although many components of the SYRCLE’s risk of bias tool are performed in experimental animal studies, they are not commonly reported. This might hamper the internal validity of the study and increase the risk of bias (ie selection, attrition, performance and detection bias). To partially overcome this problem, authors were contacted to clarify some components of this tool. Future preclinical studies should consider using the Design and Execution of Protocols for Animal Research and Treatment (DEPART) and Animals in Research: Reporting In Vivo Experiments (ARRIVE) guidelines in order to reduce variability and the risk of bias and increase translation to the human condition. In addition, these guidelines address all parameters required for complete study reporting and subsequently, improve the quality of evidence for inclusion of preclinical research in meta‐analyses and systematic reviews.

Second, the preclinical studies included in this systematic review did not resemble the clinical scenario, where only subjects diagnosed with cancer receive chemotherapy. Most experimental animal models do not take into consideration the complexity of the interactions between tumour and chemotherapy, thereby limiting the findings of the underlying molecular pathways that are activated only in the presence of chemotherapeutic agents. During cancer, the hormonal and cytokine environment is different, which might affect molecular signalling pathways. Therefore, it is difficult to generalize the results of experimental animal studies to humans.^51^ Ideally, the effect of doxorubicin on skeletal muscle tissue should be compared in tumour‐bearing and non‐tumour‐bearing animals. However, results from preclinical studies provide a controlled and valuable model to examine molecular pathways, investigate a possible dose‐response, separate the effects of doxorubicin from tumour‐induced atrophy and test potential therapeutic targets. Nevertheless, more research is needed to confirm the proposed signalling pathways in humans paving the way for potential therapeutic approaches.

Numerous studies have shown that parameters of body composition, including loss of muscle mass, muscle weakness and increases in adipose tissue are associated with unfavourable health outcomes. However, knowledge regarding muscle loss during systemic treatment and its probable clinical impact in human is lacking. To date, only two human studies evaluated the changes in body composition during the course of disease, while incorporating the effects of systemic treatment. A study by Rier et al (2018) showed that muscle attenuation (ie muscle density) significantly decreased in patients with metastatic breast cancer receiving paclitaxel.^52^ In patients treated with anthracyclines, muscle attenuation did not significantly change. The amount of muscle mass and adipose tissue remained stable during the treatment with both paclitaxel and anthracyclines. Kurk et al (2018) found that skeletal muscle mass appeared to be influenced by the intensity of palliative systemic treatment in patients with metastatic colorectal cancer.^53^ Specifically, this study reported that skeletal muscle mass loss during initial treatment with six cycles of CAPOX‐B was reversible during less intensive maintenance treatment with CAP‐B or observation. However, when a more intensive treatment was reintroduced, skeletal muscle mass decreased again. Nevertheless, it remains difficult to determine the true impact of systemic treatment on muscle in human without a valid reference population, since it is unethical to refrain from prescribing systemic treatment when it is indicated. Furthermore, other factors might be of influence as well, including decreased physical activity levels and nutritional intake. Finally, these studies depend on CT‐scans taken in routine care (ie every 9 weeks), which hampers the possibility to assess the acute effects of systemic treatment on muscle. All aforementioned caveats show the importance of preclinical studies in addition to human studies, since it allows us to determine the true impact of systemic treatment. In addition, preclinical studies enable us to investigate the underlying mechanisms as well.

### 4.2 Outlook

Treatment that concomitantly decreases protein degradation and increases protein synthesis in skeletal muscle would be most beneficial to prevent doxorubicin‐induced skeletal muscle atrophy. Nevertheless, an adequate intake of nutrients should be a prerequisite of any treatment in order to maintain or gain muscle mass.^54^ In a recent study, Hulmi and coworkers proposed blocking of activin receptor type IIb (ACVR2B) ligands by administration of soluble ligand binding domain of ACVR2B (sACVR2B‐Fc) to counteract doxorubicin‐induced skeletal muscle atrophy. They found that sACVR2B‐Fc treatment effectively prevented doxorubicin‐induced loss of muscle mass and was even able to increase muscle mass because of reduced activation of the p53‐p21‐REDD1 pathway.^36^ Furthermore, a preclinical study of Min *et al* (2015) revealed that treatment with mitochondrial‐targeted antioxidant (SS‐31) prevents the doxorubicin‐induced increase in mitochondrial ROS production and muscle atrophy.^55^ Except for pharmacological treatments, emerging evidence suggests that exercise might ameliorate the detrimental effects of doxorubicin on skeletal muscle tissue. A recent review of Powers *et al* (2019), including preclinical studies, suggests that endurance exercise training performed prior to doxorubicin treatment protects against doxorubicin‐induced skeletal muscle atrophy by the prevention of excess oxidative stress and the activation of proteolytic signalling pathways.^56^ Indeed, the beneficial effects of exercise are confirmed in human studies with several clinical trials showing that combined resistance and endurance training has positive effects on muscle strength in cancer patients undergoing chemotherapy.^57^, ^58^, ^59^ Mijwel *et al* (2018) compared the effects of different exercise regimens (moderate‐intensity aerobic combined with high‐intensity interval training vs resistance combined with high‐intensity interval training) with usual care on skeletal muscle morphology in breast cancer patients receiving chemotherapy and found that resistance combined with high‐intensity interval training resulted in a significant increase in type I muscle fibres, whereas aerobic combined with high‐intensity training counteracted a decline in type I muscle fibres.^60^ Only the resistance combined with high‐intensity interval training prevented a decline in type IIa muscle fibres. Furthermore, they showed that both exercise regimens are potent stimuli in counteracting a reduction in mitochondrial content as represented by citrate synthase activity. They also found a significant increase in protein levels of SOD2, a scavenger of ROS, in the usual care group, whereas SOD2 remained stable in both exercise groups. These findings illustrate the importance of implementing exercise programs for patients with cancer during chemotherapy to counteract the detrimental effects of chemotherapy by preserving skeletal muscle mass.

### 4.3 Conclusion

In conclusion, the findings of this meta‐analysis indicate that doxorubicin administration is associated with skeletal muscle atrophy in terms of reduced skeletal muscle weight, muscle fibre CSA and muscle strength. Furthermore, we provide an overview of how multiple pathways interact and are involved in the development of doxorubicin‐induced skeletal muscle atrophy. Reactive oxygen species, the ubiquitin‐proteasome system and autophagic markers are suggested to play a pivotal role in doxorubicin‐induced skeletal muscle atrophy. Although our knowledge with respect to the underlying mechanisms of doxorubicin‐induced muscle atrophy considerably expanded the past decade, we would like to propose that first, the precise mechanisms of chemotherapy‐induced skeletal muscle atrophy need to be carefully characterized using valid study designs, allowing for replication and comparison of studies. This would help to determine the relative contribution of each pathway involved and to identify and develop treatments that might protect against chemotherapy‐induced skeletal muscle atrophy. This first step is particularly important for chemotherapeutic agents that are in widespread clinical use. Furthermore, the effect of doxorubicin should be assessed in the context of cancer and comorbidities (eg heart failure or diabetes), since this might elicit different pathways and as a consequence different strategies might be needed to address this problem. Finally, little is known about the effects of doxorubicin on skeletal muscle nuclear epigenetics and effective biomarkers of early‐stage muscle atrophy. The latter is important to detect the loss of skeletal muscle mass at an early stage, since this might increase the effectiveness of a treatment and subsequently, the possibility to reverse this negative effect. We anticipate that if future studies head into these directions, this will provide the basis for the development of targeted counterstrategies.

## 5 MATERIALS AND METHODS

This meta‐analysis and systematic review is based on eligible, published results of human and animal experiments studying the effect of doxorubicin on skeletal muscle tissue and/or the proposed underlying mechanisms of this effect. The inclusion criteria were specified in advance. The requirements of the PRISMA (Preferred Reporting Items for Systematic Reviews and Meta‐Analyses) statement were followed (Data S1).^61^

### 5.1 Search strategy and selection of studies

A comprehensive and systematic search of the literature was conducted in PubMed, EMBASE, Web of Science and CENTRAL databases (last search performed February 5th, 2018). A Population Intervention Comparison Outcome (PICO) framework was adopted to structure the search strategy. The search strategy was developed in collaboration with an information specialist from the university library of Karolinska Institutet, Sweden. The searches were limited to animal and human studies and no time filter was applied. Only Dutch and English articles, published in peer‐reviewed journals, were considered. The detailed search strategy is provided in Data S2. Reference lists of the selected relevant papers were scrutinized to identify additional eligible studies. The citations with abstracts were uploaded into a reference database (Mendeley) and checked for duplicates. Subsequently, the citations with abstracts were uploaded into the web application Rayyan for efficient abstract screening.^62^

First, two researchers (AEH and KAB) independently assessed all titles and abstracts. Subsequently, the full‐text of all publications potentially eligible for inclusion was assessed. Discrepancies were resolved by discussion and if necessary a third reviewer (AMM) was consulted. Studies were included when they met the pre‐specified inclusion criteria: (a) design: randomized controlled trials (RCT) or controlled trials (CT); (b) population: humans, aged 18 years and older, diagnosed with a solid tumour or (non‐) tumour‐bearing animals in vivo; (c) intervention: doxorubicin administration; (d) comparison: an appropriate control group (defined as a control comparison group without tumour not receiving doxorubicin at any time point during the trial or control comparison group with tumour not receiving doxorubicin at any time point during the trial); (e) outcome: (1) quantification of the effect of doxorubicin on skeletal muscle (in terms of CSA of skeletal muscle fibres (μm^2^) or muscle mass (mg/g)) and/or (2) a description of the proposed underlying molecular pathways of this effect.

### 5.2 Risk of bias assessment

The internal validity of included studies was independently assessed by two researchers (AEH and KAB) using SYRCLE’s risk of bias tool.^63^ This tool is based on the Cochrane Collaboration risk of bias tool^64^ and has been adapted to aspects of bias that play a role in experimental animal studies. The tool contains 10 entries, which are related to 6 types of bias: selection bias, performance bias, detection bias, attrition bias, reporting bias and “other” biases. The score “yes” indicates a low risk of bias, whereas “no” indicates a high risk of bias and “?” an unclear risk of bias.

In order to give a representative and realistic overview of the internal validity of all included studies, authors of the included studies were contacted and asked to clarify some components of this tool if not reported in the publication. In addition to SYRCLE’s risk of bias tool, five entries were added to overcome the problem of judging too many items as unclear risk of bias. Therefore, data on five study quality indicators were extracted: (a) any measure of randomization; (b) any measure of blinding; (c) temperature regulation; (d) ethical approval and (e) conflict of interest statement. For these additional items, a “yes” indicates reported, and a “no” indicates unreported.

### 5.3 Data extraction

One investigator (AEH) extracted data. Information from papers was extracted related to: study aim, study design, type of study population, sample size, demographical characteristics and outcome measures. The primary outcome, the effect of doxorubicin on skeletal muscle, was measured as skeletal muscle fibre CSA (μm^2^) and/or as muscle weight of soleus, gastrocnemius, tibialis anterior or extensor digitorum longus muscle. Group averages (mean) with corresponding standard deviation (SD) or standard error (SE), and number of animals per group (*n*) were extracted for both outcomes, if available. The authors were contacted to request these point estimates and measures of variability, if the results were presented incomplete or graphically only. In case of no response, graphically presented data were converted to numerical data by eyeballing.

### 5.4 Data‐analysis

Data were synthesized to compare the outcomes, skeletal muscle fibre CSA and skeletal muscle weight, for subjects receiving doxorubicin and subjects not receiving doxorubicin. Percentage change in the outcome was calculated (eg mean muscle weight in the doxorubicin‐treated group minus mean muscle weight in the vehicle control group, divided by mean muscle weight in the vehicle control group) with corresponding 95% confidence interval (CI). The percentage change in the outcome across all individual studies was pooled to obtain an overall percentage change and 95% CI. For the outcome skeletal muscle weight, subgroup analyses were conducted to assess the effect of doxorubicin on specific limb muscles. Heterogeneity was quantified by I^2^. Since significant heterogeneity was present, a random effects model was applied to account for variation between studies. Data were analysed using R version 3.5.0.

Regarding the underlying molecular mechanisms of doxorubicin‐induced skeletal muscle atrophy, a qualitative summary of the available evidence was produced.

## CONFLICT OF INTEREST

All authors declare that they have no conflict of interest.
