# Supplementary material for: Doxorubicin‐induced skeletal muscle atrophy: Elucidating the underlying molecular pathways
Source: Acta Physiol (Oxf). 2019 Oct 31;229(2):e13400. doi: 10.1111/apha.13400 (PMC7317437; doi:10.1111/apha.13400)
Supplement: Supplementary file 2 [file APHA-229-e13400-s002.docx]

**Additional file 2 – Full search strategies

PICO-defined research question**

*Aims*

1. To quantify the effect of doxorubicin on skeletal muscle tissue.
2. To evaluate the findings of studies that sought to determine the molecular pathways leading to doxorubicin-induced muscle atrophy in both human and animal models.

*Study design*

Randomized controlled trials, non-randomized controlled trials examining underlying mechanisms of chemotherapy-induced muscle atrophy.

*Participants/population*

• Men and/or women, aged 18 years and older, diagnosed with a solid tumor

• Rodents (with/without cancer)

*Intervention*

• Doxorubicin

*Comparators/Controls*

Note: Muscle atrophy has to come from doxorubicin alone rather than from other contributors. Therefore, a true control is a requirement for inclusion in this systematic review.

• True control: control comparison group (without tumor, healthy subjects) not
 receiving doxorubicin at any time point during the trial.

• True control: control comparison group (with tumor) not receiving doxorubicin at
 any time point during the trial.

*Outcome*

1. Quantification of doxorubicin-induced muscle atrophy (i.e., cross-sectional area of skeletal muscle fiber or muscle weight) AND/OR
2. Underlying molecular pathways of doxorubicin-induced muscle atrophy.

| **PubMed** | |
| --- | --- |
| **Chemotherapy** | Doxorubicin[MeSH Terms] OR doxorubicin[Title/Abstract] |
| **Muscle atrophy** | Muscular atrophy[MeSH Terms] OR muscle weakness[MeSH Terms] OR cachexia[MeSH Terms] OR wasting syndrome[MeSH Terms] OR muscle atrophy[Title/Abstract] OR muscle loss[Title/Abstract] OR muscle wast*[Title/Abstract] OR muscular atrophy[Title/Abstract] OR muscle weakness[Title/Abstract] OR cachexia[Title/Abstract] OR wasting syndrome[Title/Abstract] OR muscle degeneration{Title/Abstract] OR amyotrophy[Title/Abstract] OR amyotrophia[Title/Abstract] |

| **EMBASE** | |
| --- | --- |
| **Chemotherapy** | 'doxorubicin'/exp OR 'doxorubicin*':ti,ab |
| **Muscle atrophy** | 'muscle atrophy'/exp OR 'muscle weakness'/exp OR 'cachexia'/exp OR 'wasting syndrome'/exp OR 'muscle atrophy':ti,ab OR 'muscle weakness':ti,ab OR 'cachexia':ti,ab OR 'wasting syndrome':ti,ab OR 'muscle wast*':ti,ab OR 'muscle degeneration':ti,ab OR 'amyotrophy':ti,ab OR 'amyotrophia':ti,ab OR 'muscular atrophy':ti,ab |
| **Study design** | 'animal experiment'/exp OR 'animal model'/exp OR 'clinical trial'/exp OR 'intervention study'/exp OR 'control group'/exp OR 'randomization'/exp OR 'animal experiment':ti,ab OR 'animal model':ti,ab OR 'clinical trial':ti,ab OR 'intervention study':ti,ab OR 'control group':ti,ab OR 'randomization':ti,ab |

| **Web of Science** | |
| --- | --- |
| **Chemotherapy** | TOPIC: (doxorubicin) |
| **Muscle atrophy** | (((((((((TOPIC: (muscle atrophy) *OR* TOPIC: (muscular atrophy)) *OR* TOPIC: (muscle loss)) *OR* TOPIC: (muscle wast*)) *OR* TOPIC: (muscle weakness)) *OR* TOPIC: (muscle degeneration)) *OR* TOPIC: (cachexia)) *OR* TOPIC: (wasting syndrome)) *OR* TOPIC: (amyotrophy)) *OR* TOPIC: (amyotrophic)) |

| **CENTRAL** | |
| --- | --- |
| **Chemotherapy** | "doxorubicin" |
| **Muscle atrophy** | "muscle atrophy" or "muscular atrophy" or "muscle loss" or "muscle weakness" or muscle wasting or "muscle degeneration" or "cachexia" or "wasting syndrome" or "amyotrophy" or "amyotrophia" |
