# Supplementary material for: Doxorubicin‐induced skeletal muscle atrophy: Elucidating the underlying molecular pathways
Source: Acta Physiol (Oxf). 2019 Oct 31;229(2):e13400. doi: 10.1111/apha.13400 (PMC7317437; doi:10.1111/apha.13400)
Supplement: Supplementary file 3 [file APHA-229-e13400-s003.docx]

**Additional file 3 – Study quality and risk of bias**

| **Study** | **Any randomization** | **Any blinding** | **Sample size calculation** | **Temperature regulation** | **Ethical approval** | **Conflict of interest statement** | **Random group allocation** | **Groups similar at baseline** | **Blinded group allocation** | **Random housing** | **Blinded intervention** | **Random outcome assessment** | **Blinded outcome assessment** | **Reporting of drop-outs** | **Other biases** |
| --- | --- | --- | --- | --- | --- | --- | --- | --- | --- | --- | --- | --- | --- | --- | --- |
| **Dirks-Naylor (2013)** | **Y** | **N** | **N** | **Y** | **Y** | **Y** | **H*** | **?** | **H*** | **H*** | **H*** | **?** | **H*** | **H*** | **L** |
| **Gilliam (2009)** | **N** | **N** | **N** | **N** | **Y** | **N** | **H*** | **?** | **H*** | **L*** | **?** | **?** | **?** | **L*** | **L** |
| **Gilliam (2011)** | **N** | **N** | **Y** | **N** | **Y** | **N** | **H*** | **?** | **H*** | **L*** | **?** | **?** | **?** | **L*** | **L** |
| **Gilliam (2011)** | **N** | **N** | **N** | **N** | **Y** | **Y** | **H*** | **?** | **H*** | **L*** | **?** | **?** | **?** | **L*** | **L** |
| **Gilliam (2013)** | **N** | **N** | **N** | **Y** | **Y** | **N** | **L*** | **L*** | **L*** | **L*** | **H*** | **?** | **H*** | **L*** | **L** |
| **Gilliam (2016)** | **N** | **N** | **N** | **Y** | **Y** | **Y** | **L*** | **L*** | **L*** | **L*** | **H*** | **?** | **H*** | **L*** | **L** |
| **Grabowiecki (2015)** | **N** | **N** | **Y** | **N** | **Y** | **N** | **L*** | **?** | **L*** | **L*** | **L*** | **?** | **L*** | **L*** | **L** |
| **Huang (2016)** | **N** | **N** | **N** | **Y** | **Y** | **Y** | **?** | **?** | **?** | **?** | **?** | **?** | **?** | **?** | **H** |
| **Hulmi (2017)** | **Y** | **N** | **N** | **Y** | **Y** | **Y** | **?** | **?** | **?** | **L** | **?** | **?** | **?** | **?** | **L** |
| **Hydock (2011)** | **Y** | **N** | **N** | **N** | **Y** | **N** | **?** | **?** | **?** | **?** | **?** | **?** | **?** | **?** | **L** |
| **Kavazis (2014)** | **Y** | **N** | **N** | **N** | **Y** | **Y** | **?** | **?** | **?** | **?** | **?** | **?** | **?** | **?** | **L** |
| **Lima Junior (2016)** | **Y** | **N** | **N** | **Y** | **Y** | **Y** | **?** | **?** | **?** | **?** | **?** | **?** | **?** | **?** | **L** |
| **Min (2014)** | **y** | **N** | **N** | **Y** | **Y** | **N** | **?** | **?** | **?** | **L** | **?** | **?** | **?** | **?** | **L** |
| **Min (2015)** | **Y** | **N** | **N** | **N** | **Y** | **Y** | **L*** | **L*** | **L*** | **L*** | **L*** | **?** | **L*** | **L*** | **H** |
| **Nissinen (2016)** | **Y** | **N** | **N** | **Y** | **Y** | **N** | **L*** | **L*** | **L*** | **L*** | **L*** | **?** | **L*** | **?** | **L** |
| **Smuder (2011)^a,b^** | **Y** | **N** | **N** | **N** | **Y** | **Y** | **L*** | **L*** | **L*** | **L*** | **L*** | **?** | **L*** | **L*** | **L** |
| **Yu (2014)** | **Y** | **N** | **N** | **N** | **Y** | **Y** | **L*** | **L*** | **L*** | **L*** | **H*** | **?** | **L*** | **L*** | **L** |
| **Yu (2015)** | **Y** | **N** | **N** | **N** | **Y** | **N** | **L*** | **L*** | **L*** | **L*** | **H*** | **?** | **L*** | **L*** | **L** |
| **Zima (2011)** | **N** | **N** | **N** | **Y** | **N** | **N** | **?** | **L** | **L** | **?** | **?** | **?** | **?** | **?** | **L** |
| **Y = Reported, N = Not reported, ? = Unclear risk of bias, L = Low risk of bias, H = High risk of bias.**  *** The risk of bias was clarified after contacting the investigators.** | | | | | | | | | | | | | | | |
